# Hexagonal Boron Nitride assisted transfer and encapsulation of large area CVD graphene

**DOI:** 10.1038/srep30210

**Published:** 2016-07-22

**Authors:** Viktoryia Shautsova, Adam M. Gilbertson, Nicola C. G. Black, Stefan A. Maier, Lesley F. Cohen

**Affiliations:** 1 Blackett Laboratory, Imperial College, Prince Consort Rd, London SW7 2BZ, UK; 2 National Physical Laboratory, Hampton Rd, Teddington, Middlesex TW11 0LW, UK

## Abstract

We report a CVD hexagonal boron nitride (hBN-) assisted transfer method that enables a polymer-impurity free transfer process and subsequent top encapsulation of large-area CVD-grown graphene. We demonstrate that the CVD hBN layer that is utilized in this transfer technique acts as a buffer layer between the graphene film and supporting polymer layer. We show that the resulting graphene layers possess lower doping concentration, and improved carrier mobilities compared to graphene films produced by conventional transfer methods onto untreated SiO_2_/Si, SAM-modified and hBN covered SiO_2_/Si substrates. Moreover, we show that the top hBN layer used in the transfer process acts as an effective top encapsulation resulting in improved stability to ambient exposure. The transfer method is applicable to other CVD-grown 2D materials on copper foils, thereby facilitating the preparation of van der Waals heterostructures with controlled doping.

Owing to its unique mechanical^1^, electronic^2^ and optical^3^ properties, graphene has attracted intense interest as a promising material for a range of technological applications including nanoelectronics and photonics. Recent advances in graphene synthesis and device fabrication techniques have stimulated further interest. In particular, chemical vapour deposition (CVD) growth of graphene on copper substrates has attracted considerable attention due to its potential for producing high-quality and large-area graphene films suitable for scalable applications^4^,^5^. However, the necessity of a subsequent transfer process onto appropriate substrates for device fabrication, and those fabrication steps themselves, introduce a myriad of potential pitfalls in attaining high quality device properties. Currently, the most common transfer methods rely on polymer layers deposited on top of the graphene (for example PMMA^6^, polystyrene^7^, polybutadiene^8^) to provide mechanical support after etching away the growth substrate. However, polymer residues left behind due to incomplete removal of the support layer are the dominant source of extrinsic doping reported in CVD graphene devices^9^.

The robustness of graphene during fabrication processes and the long-term stability of graphene devices are additional key issues that must be resolved in order to make graphene commercially applicable. Improved stability is attainable by encapsulating graphene devices with a protection layer. A number of approaches have been reported employing a variety of encapsulation materials including metal oxides (Al_2_O_3_^10^,^11^,^12^, TiO_2_^13^), polymer layers^14^, organic semiconductors^15^ and self-assembled monolayers (SAMs)^16^. However, these processes are frequently incompatible with generic fabrication techniques and degrade the electronic properties of graphene, resulting in comparably low carrier mobilities in the range of 1000–5000 cm^2^ V^−1^ s^−1^. Recent attention has been devoted to graphene encapsulation using hexagonal boron nitride (hBN) layers, a lattice-matched insulating 2D material with atomically smooth surfaces that has enabled near phonon-limited room temperature carrier mobilities of 140,000 cm^2^ V^−1^ s^−1^ to be realised in exfoliated hBN/graphene/hBN heterostructures^17^,^18^. So far, this method has been widely reported for exfoliated hBN material, with limited attention to scalability. In this letter, we demonstrate a CVD hBN-assisted transfer method for large area CVD graphene that eliminates the direct contact between graphene and polymer layers and results in full device encapsulation. The encapsulated devices show significantly lower doping, higher mobility and greater stability to ambient exposure than graphene devices prepared by other routes.

## Results and Discussion

**Figure 1:**
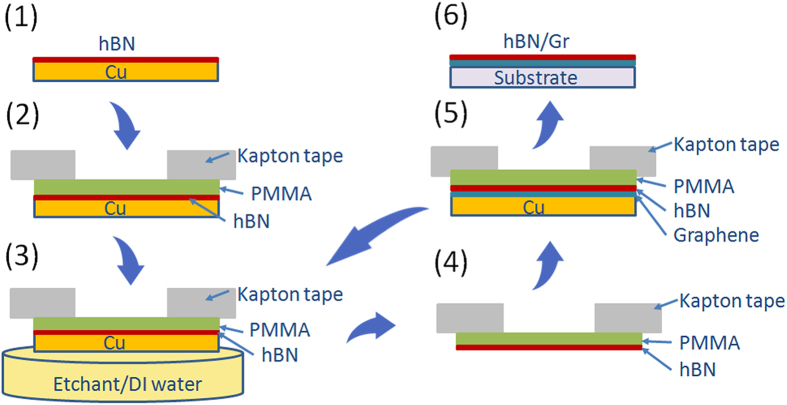
Schematic of the hBN-assisted transfer process. See Methods section for more details.

Figure 1 presents a schematic of the hBN-assisted transfer (AT) technique (for details, see Methods). The process starts as a conventional transfer method with the stamp applied to the PMMA/hBN/Cu stack. After Cu etching and DI water rinsing, the sample is dried and then directly applied to the graphene/Cu foil. By these means, a clean interface between the hBN and graphene layer is created. The number and order of stacked 2D layers is in principle unlimited. In the case of a conventional transfer technique the accumulation of PMMA residues is observed with every next transferred layer, significantly limiting the final properties of the structure^19^. To provide comparable results, in this work we consider only one hBN-Gr stack, which is applied to the SiO_2_/Si substrate by a scooping technique (AT-hBN/Gr/SiO_2_ sample), but the final result can be further improved by dry transfer and full encapsulation with hBN.

The novel transfer method is compared to the conventional, and improved wet transfer (WT) techniques. It has been demonstrated that SiO_2_ surface passivation techniques such as SAMs or hBN buffer layers significantly improve the electrical properties of CVD graphene^20^,^21^,^22^,^23^, which are usually limited in graphene/SiO_2_ structures by charged surface states and impurity scattering^24^,^25^,^26^, surface optical phonons^26^,^27^ and substrate roughness^28^,^29^,^30^. Thus, an AT-hBN/Gr/SiO_2_ sample is compared to three samples prepared using the WT technique differing only in the substrate treatment, including untreated SiO_2_ (WT-Gr/SiO_2_), SAM-modified SiO_2_ (WT-Gr/SAM) and hBN covered SiO_2_ (WT-Gr/hBN). For the SAM modification, an octadecyltrichlorosilane (ODTS) layer was utilized due to its higher efficiency of screening the charge impurities present on SiO_2_ layer^20^,^31^. It should also be noted that surface engineering provides additional benefits by improving the graphene adhesion, which is particularly important in the dry transfer case (Supplementary Fig. S1).

Optical microscopy images of the transferred graphene films confirm that regardless of the target substrate, the WT method results in continuous films with low density of defective structures such as tears and cracks (Supplementary Fig. S2). We note that in the AT process the target Gr/Cu substrate is no longer flat as is the case for WT onto SiO_2_, and a topological mismatch of PMMA/hBN and Gr/Cu interfaces is likely to be present because the PMMA film conforms to the morphology of the underlying copper substrate of the first hBN layer^32^. As a result, an increased number of wrinkles and structural defects may be expected. Surprisingly, we find that the AT method results in the same quality of graphene film as found in the WT samples. This result highlights the necessity and effectiveness of the polymer relaxation step in the AT method. During this step the PMMA is heated above the glass transition temperature to increase the contact with the target substrate and, in case of a Graphene/Cu substrate, to adopt the new copper surface morphology.

**Figure 2:**
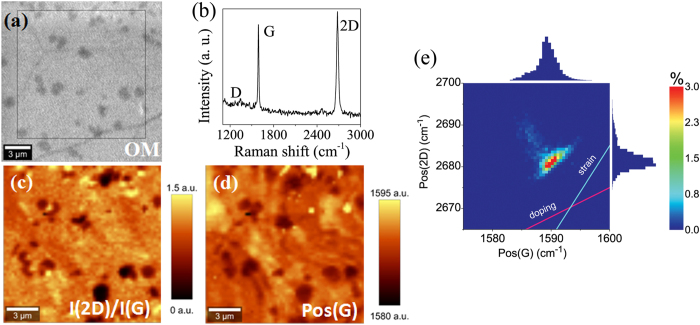
Typical characterization results for graphene wet transferred on bare Si/SiO_2_. (**a**) Optical microscopy image with the map area defined (dark areas are multilayer regions). (**b**) Raman spectrum of graphene with the main peaks labelled. Micro-Raman images of the I(2D)/I(G) ratio (**c**) and G-peak position (**d**). (**e**) 2D histogram describing correlation between positions of the 2D- and G-peaks.

**Figure 3:**
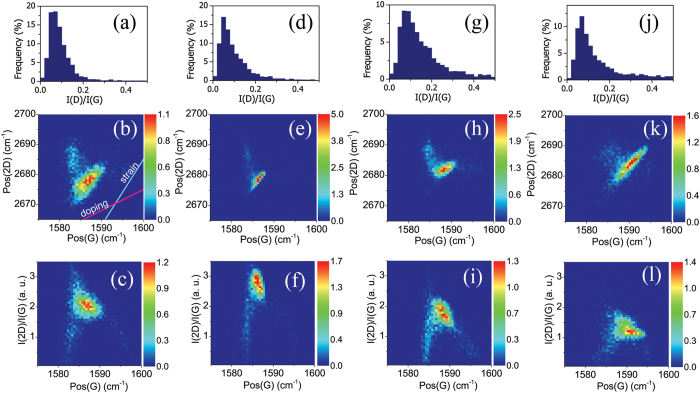
Raman spectroscopy results. The samples are transferred using a wet transfer technique on untreated SiO_2_ (**a–c**), SAM- (**d–f**) and hBN- (**g–i**) covered SiO_2_ and using hBN-assisted transfer (**j–l**). The first row describes defect density in the graphene films by presenting the I(D)/I(G) ratio. The second and third rows are 2D histograms describing correlations between the 2D- and G-peak positions and the I(2D)/I(G) ratio and the G-peak position, respectively.

The crystalline quality of the graphene films was analysed by Raman spectroscopy, as shown in Figs 2 and 3. Since strong spatial variations in the Raman spectra are usually observed over the transferred graphene film^33^, areas of 15 × 15 μm were mapped out and analysed to provide a sufficient statistical description of the overall graphene quality. Raman data is presented in the form of 2D histograms as shown in Fig. 2e, to simplify the comparison between graphene samples. Raman spectroscopy provides a significant amount of information about the graphene material, such as the number of layers, doping, defects, strain, disorder, chemical modifications and edges^34^,^35^,^36^,^37^. Here, we concentrate on defect, doping and strain analysis. A representative Raman spectrum of CVD graphene shown in Fig. 2b displays the well-known D, G and 2D peaks. The D-peak defined by the breathing modes of the carbon rings is active only in the presence of defects^35^, making it an ideal marker for defect concentration analysis in graphene samples. An independent analysis of the doping and strain present in graphene films is often problematic, since both processes affect the positions of the G and 2D peak^37^,^38^,^39^,^40^,^41^,^42^, Pos(G) and Pos(2D), respectively. However, the doping and strain-induced Raman shifts ∆Pos(2D) and ∆Pos(G) exhibit distinct correlations according to ∆Pos(2D) ≈ γ ∆Pos(G), where γ is a correlation coefficient for the specific process^38^,^39^,^43^. For strained graphene, values for γ in the range of 2.2–2.8 have been demonstrated both experimentally and theoretically^38^,^39^,^43^, while for the electrically doped graphene the value of γ ≈ 0.75 was established^43^. Therefore, using a pair of guide curves with appropriate slopes, it is possible to predict the contribution from both strain and doping variations in the graphene films simultaneously (Fig. 2e).

Consistent with optical microscopy, the obtained micro-Raman maps show uniform coverage of the graphene films transferred using both conventional WT and the presented AT methods (Supplementary Fig. S2). Raman spectroscopy confirms the presence of multilayer areas and occasional residue particles on the graphene film, which appear as dark regions in the I(2D)/I(G) map (Fig. 2). The residue particles can be separated from multilayer areas by analyses of the Pos(G) map. The residues are presented by bright regions due to increased doping of the graphene, while multilayer areas remain dark due to screening of surface charges on the substrate^44^. Representative Raman spectra for the samples are shown in the Supplementary information Fig S3. It should be noted that the intensity of the Raman peak for the hBN monolayer observed at 1366 cm^−1^ is 50 times weaker than for the G peak of graphene under same measurement conditions^45^ and, thus, under 0.5 s integration time used in the current study, the hBN peak is unresolved and requires much longer integration time (>60 s). The transferred graphene films demonstrate low defect concentration (Fig. 3), where the I(D)/I(G) ratio is typically less than 0.2^35^. Interestingly, the WT-Gr/hBN sample has a slightly higher defect concentration, which may be attributed to the presence of polymer residues left on the hBN surface after the first transfer and possibly by multilayer areas of the CVD hBN. Notably, the AT method (AT-hBN/Gr/SiO_2_ sample) does not introduce significant defects and results in a defect density even lower than that detected for the WT-Gr/hBN sample.

The 2D histograms for Pos(G) and Pos(2D) are shown in Fig. 3(b,f,i). To interpret the distributions, we compare the observed correlations with the expected variations for strain and doping as indicated by the guide curves shown in Fig. 3b (and as set out in the example shown in Fig. 2 and the associated discussion). From the trends observed in the 2D histograms (see also Supplementary Fig. S4), it is possible to conclude that the variations in Pos(G) and Pos(2D) are largely due to strain present in all samples, which is consistent with previous results for exfoliated graphene samples^43^. For the samples WT-Gr/hBN and AT-hBN/Gr/SiO_2_ where graphene is in contact with the hBN film, a pronounced upshift of the Pos(2D) is observed, indicating increased strain. Sample AT-hBN/Gr/SiO_2_ exhibits an elongated distribution, providing further evidence of increased strain variation, as expected from the AT technique. In contrast, the conventional WT-Gr/SiO_2_ sample exhibits correlations between Pos(G) and Pos(2D) that are consistent with doping variations in the sample. This is attributed to the fact that the graphene film is in direct contact with SiO_2_, which introduces doping variation due to the presence of charged surface states and impurities^24^,^25^,^26^. Accordingly, by introducing the ODTS buffer layer (WT-Gr/SAM), we see a significant reduction in the doping variations (Fig. 3c), which is in a good agreement with previous results for graphene transferred on SAM-modified substrates^20^,^31^. The monolayer of ODTS effectively minimizes the density of unintentional dopants and charge traps on the substrate. The same mechanism is expected for the WT-Gr/hBN sample, where the hBN layer should screen the underlying SiO_2_ substrate. However, since the CVD hBN layer was transferred using the conventional WT method, polymer residues present on the surface introduce additional doping variations in the WT-Gr/hBN sample. Notably, the AT-hBN/Gr/SiO_2_ sample demonstrates lower doping variations even though the graphene film is in direct contact with SiO_2_, which is consistent with the absence of surface polymer residues. The correlations between the Pos(G) and I(2D)/I(G) are shown in the 2D histograms of Fig. 3(c,f,i,l). Of the WT samples, the WT-Gr/SAM exhibits the largest average I(2D)/I(G) ratio, indicating the low doping. The high strain present in the AT-hBN/Gr/SiO_2_ sample makes the doping analysis difficult.

The material quality was further analysed by examining the electrical properties of graphene FET devices in terms of the charge neutrality point and carrier mobility (see Methods section for more details on the fabrication process). The charge neutrality point, or the Dirac point (V_D_), defined as the conductivity minimum, provides direct information about the magnitude of the extrinsic doping and facilitates comparison between the different transfer techniques. Although carrier mobility is another important parameter for the material quality characterization, device length scale is important. In the current study, we have chosen to make the devices using a shadow mask technique. The advantage of this technique is that it avoids exposing the graphene to additional polymer residues for example during a photoresist or ebeam resist step, allowing us to isolate extrinsic doping induced by the transfer method alone. Additionally, shadow masking prevents graphene removal and delamination during lift off process^46^. However, the technique generally results in large area devices (of the order of 100 × 1000 microns in our case), and at these length scales the mobility value provides a useful measure of the density of defects present in the CVD graphene devices (such as multilayers, grain boundaries, wrinkles and occasional tears) but does not reflect the defect free mobility value. In order to reach the intrinsic mobility, submicron devices would be necessary. As doping due to the transfer method is the focus of this study, the intrinsic mobility measurements are out of scope of the current work. Nevertheless, the extracted mobility in these large area devices remains a useful parameter, as it allows direct comparison of additional extrinsic defects introduced by the transfer techniques.

**Figure 4:**
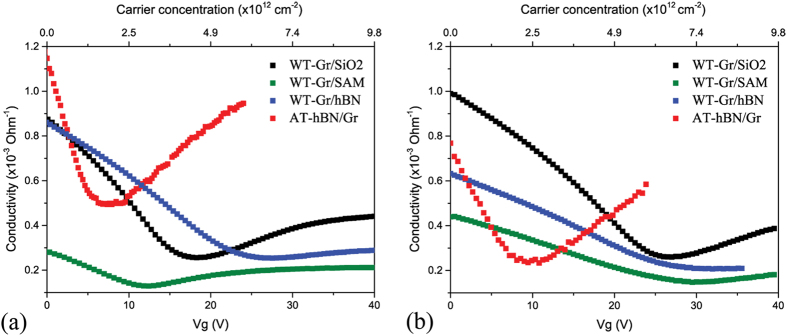
Conductivity of the graphene FETs obtained after wet transfer on untreated SiO_2_, SAM- and hBN- covered SiO_2_ and from hBN-assisted transfer after preparation (**a**) and after 1 month storage in ambient conditions (**b**).

In Fig. 4(a) we show the dependence of the two-terminal conductivity on the gate voltage. The comparative results from Fig. 4 are summarized in Table 1. It should be noted, that by studying multiple devices we can comment on the reproducibility of our results (see Supplementary Fig. S5 and Table S1). We find that for any one device type V_D_ is a rather robust, reproducible to within ±10%, whereas the hole carrier mobility varies by as much as ±25%. Comparing different samples it is clear that the charge neutrality point shows significant variation, which is consistent with doping analysis of Raman spectroscopy results. (Note that the absolute value of the conductivity minimum is dependent on the contact resistance and is therefore not greatly informative). For the sample WT-Gr/SAM, the V_D_ is located at lower gate voltages compared to WT-Gr/SiO_2_ implying reduced p-type doping, consistent with the passivation of the SiO_2_ surface by the SAM. In contrast, the hBN buffer layer (WT-Gr/hBN) appears to have a negative effect, displaying a V_D_ upshift compared to the WT-Gr/SiO_2_ sample, indicating an increased p-type doping, consistent with the presence of polymer residues on the underlying hBN surface. The lowest doping is observed for the hBN-assisted transfer (AT-hBN/Gr/SiO_2_) which exhibits a V_D_ ~ 7 V, significantly downshifted compared to WT-Gr/SiO_2_. Since the graphene film is still in direct contact with the SiO_2_ substrate, this result can be attributed directly to the absence of polymer residues on the graphene surface. From analysis of the V_D_ we deduce that the absence of polymer residues results in a 2.7 × 10^12^ cm^−2^ reduction in the doping level (see Table 1).

In order to assess the long-term stability of the samples produced from different transfer methods, the electrical properties were measured again after the samples had been stored for one month under ambient conditions. Results are shown in Fig. 4(b). The change in the absolute conductivity value is a combination of the change of carrier doping in the graphene film and any changes to the contact resistance, which is also captured by two terminal electrical measurements. Significant degradation of the electrical properties is observed for the unprotected graphene samples WT-Gr/SiO_2_, WT-Gr/SAM and WT-Gr/hBN, as evidenced by a general increase of V_D_ (see Table 1). Although in general, the hydrophobic SAM should be less susceptible to water or oxygen adsorbents^20^,^21^,^22^,^23^, in this study we find that the WT-Gr/SAM sample shows the most significant V_D_ increase with time. However, to the best of our knowledge the aging characteristics for graphene FET based on SAMs have not been previously reported. The encapsulated AT-hBN/Gr/SiO_2_ sample demonstrates a high stability with a modest increase of the V_D_ by 2.6 V only. This is in good agreement with previous reports of the negligible environmental sensitivity for an exfoliated hBN-graphene-hBN sandwich structure based on exfoliated material^47^.

A lower bound for the extrinsic mobilities of the graphene films was extracted from the slope near the Dirac point using the following equation:


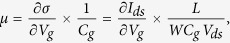


where *L* and *W* are the channel length and width, respectively, and *V*_*ds*_ is the drain-source voltage. The capacitance *C*_*g*_ = 3.93*10^−8^ F cm^−2^ is calculated using 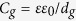, where *ε* is the dielectric constant of SiO_2_ (~4), *ε*_*0*_ is the permittivity of free space and *d*_*g*_ is the thickness of SiO_2_ (90 nm). This method provides results comparable to those reported previously using a similar calculation of the graphene mobility from two-point FET measurements^20^,^31^,^48^,^49^. The extracted electron and hole mobilities from devices shown in Fig. 4 are listed in Table 1. A hole carrier mobility of around 1000 cm^2^ V^−1^ s^−1^ is obtained for the Gr-SiO_2_ sample, which is consistent with literature values^31^,^50^. Moreover, the conductivity curve demonstrates a significant asymmetry in the hole and electron mobilities (*μ*_*h*_*/μ*_*e*_ ~ 3.2), which can be attributed to the presence of charge trap states on the SiO_2_ substrate^51^ or drift in the charged impurities from the substrate^52^. As stated above, the statistical variation in the hole mobility of any one device type is of the order of 25%, nevertheless, it is interesting to note that even taking this into account the devices produced from WT-Gr/SAM structure exhibit a reduced mobility compared to those of WT-Gr/SiO_2_. This is perhaps surprising, since the SAM is expected to passivate reactive sites such as silanol groups at the SiO_2_ surface^20^. However, we note that a significant variation in the carrier mobilities of Gr/SAM samples has been reported in the literature^20^,^31^, which suggests the outcome is quite susceptible to device transfer handling and processing methodology for this particular device type. We point out that in the WT process, graphene films deposited on a hydrophobic substrate can delaminate due to the high contact angle between DI water and the substrate, resulting in formation of macro-cracks. The WT-Gr/SAM sample demonstrates a higher hole to electron mobility ratio of *μ*_*h*_*/μ*_*e*_ ~ 2.2 indicating that the ODTS layer screens the charged impurities from the adsorbates on the SiO_2_ surface. In the case of the WT-Gr/hBN sample, while the hole mobility remains quite high, the hole/electron mobility imbalance is much stronger with *μ*_*h*_*/μ*_*e*_ ~ 9 providing further evidence of the presence of polymer dopants on the hBN surface. Finally, we find that for the AT-hBN/Gr/SiO_2_ sample the electron and hole mobilities are increased several-fold (even taking into account reproducibility across devices) compared to the standard WT-Gr/SiO_2_ sample. Noticeably, the asymmetry of the electron and hole mobilities remains high (*μ*_*h*_*/μ*_*e*_ ~ 3.7) due to direct contact of the graphene film with the SiO_2_ substrate.

After 1 month of storage in ambient conditions significant decrease of the carrier mobilities is observed for the unprotected graphene samples WT-Gr/SiO_2_, WT-Gr/SAM and WT-Gr/hBN (Table 1). Interestingly, whilst the doping level remains low for the encapsulated AT-hBN/Gr/SiO_2_ sample, there is degradation of the carrier mobility, particularly the hole mobility, in this sample. Mobility degradation can be attributed to direct contact of the graphene film with the underlying SiO_2_ which attracts dipolar adsorbates and, thus, facilitates sample aging^53^. We note that similar air stability has been recently reported for CVD graphene encapsulated with a combination of parylene-C and aluminum thin film^55^. Whilst in that reference, the device mobility demonstrates only a 5% decrease, the initial carrier mobility is 3-fold smaller compared to the encapsulated AT-hBN/Gr/SiO_2_ sample discussed in the current study.

## Conclusion

In conclusion, we have presented an hBN-assisted transfer method that enables a polymer free transfer and subsequent encapsulation of CVD-grown large-area graphene. Utilizing this method, we demonstrated graphene FET devices with a much lower doping concentration and improved carrier mobilities compared with the results of the conventional transfer method for untreated SiO_2_, SAM-modified and hBN covered SiO_2_. Moreover, the top hBN layer used in the transfer process provides excellent encapsulation, resulting in greater stability to ambient exposure. We show that the transfer method we describe facilitates the development of high quality and large area hBN encapsulated graphene films. Moreover, we anticipate its applicability to other CVD-grown 2D materials enabling the preparation of van der Waals structures with atomically clean interfaces.

## Methods

### Graphene wet transfer (WT) technique

Monolayer graphene/Cu (25 μm) and hBN/Cu (25 μm) foils were purchased from Graphenea and Graphene Supermarket, respectively. Graphene/Cu or hBN/Cu layers were first covered with PMMA A4 495 by spin coating at 3500 rpm for 60 s and curing at room temperature overnight. Graphene or hBN present on the bottom side of the foils were removed using an O_2_-plasma treatment at 100 W during 2 mins. The exposed Cu foil is then etched in an ammonium persulfate solution (15 g/L) followed by a thorough rinse in DI water. The floating PMMA/graphene or PMMA/hBN stack was scooped out using the target substrate. After drying, the sample was annealed at 180 °C for 30 mins. Finally, the PMMA was removed in acetone followed by IPA rinse. Three samples were prepared using the conventional wet transfer (WT) technique differing only in the substrate treatment, including untreated SiO_2_ (WT-Gr/SiO_2_), SAM-modified SiO_2_ (WT-Gr/SAM) and hBN covered SiO_2_ (WT-Gr/hBN). For the SAM modification, an octadecyltrichlorosilane (ODTS) layer was utilized due to its higher efficiency of screening the charge impurities present on SiO_2_ layer^20^,^31^. The ODTS layer was deposited by O_2_-plasma activation of the SiO_2_ and spin casting from toluene solution^54^. In the case of WT-Gr/hBN sample, the CVD hBN film is transferred first using the same WT technique which is outlined above.

### Graphene hBN-assisted transfer (AT) technique

The hBN/copper foil was coated with a relatively thick layer of PMMA (7% at 2500 rpm for 60 s). A frame of kapton tape was then applied to the PMMA/hBN/Cu stack to facilitate the dry transfer. After Cu etching and rinsing, the PMMA/hBN stack was removed from the DI water and dried at 60 °C for 1 hour. The sample was then ready for application directly to the target substrate, which in this case was the graphene/Cu foil. By these means a clean interface between hBN and graphene layer was created. After annealing at 180 °C for 30 mins, Cu etching and rinsing steps were repeated. The number and order of stacked 2D layers is in principle unlimited. To provide comparable results, in this work we limit ourselves to one hBN-Gr stack, which was applied to the SiO_2_ using the WT procedure (AT-hBN/Gr/SiO_2_ sample).

### Transport measurements

The electrical properties of the transferred graphene films were examined in a field-effect transistor (FET) geometry. For the WT samples, top-contact Cr (5 nm)/Au (40 nm) electrodes were thermally evaporated through shadow masks directly onto graphene films to avoid further contact with polymers such as photoresist. For the hBN-assisted sample, photolithography can be used due to the protection provided by the top layer hBN. In this case, the edge-contacts were fabricated using a two-step photolithography process^17^. The first step defined the device shape by removing the residual hBN/Gr material by O_2_-plasma treatment, while the second step provided the necessary pattern for contact deposition (Cr (5 nm)/Au (40 nm)). Graphene FET channel width and length were 100 and 1000um, respectively. The transfer characteristics of the graphene FETs were analyzed using a Keithley 2634B semiconductor parameter analyser unit under ambient environment with a two-point measurement for source and drain electrodes.

### Raman spectroscopy

Raman spectroscopy measurements were performed using a confocal scanning Raman microscope (WiTec Alpha300 system) in backscattering geometry with 532 nm excitation and a 100 × objective (0.9 NA). An incident laser power of 1 mW was used to avoid laser induced heating of graphene samples. Measurements were performed at room temperature under ambient conditions, with an accumulation time of 0.5 s, a spectral resolution of 3 cm^−1^ and lateral resolution of 0.3 μm. Areas of 15 × 15 μm were mapped out and analysed to provide a sufficient statistical description of the overall graphene quality, since strong spatial variations in the Raman spectra are usually observed over the transferred graphene film^33^. Additionally, we present data in the form of 2D histograms to simplify the comparison between graphene samples in terms of the carrier concentration and strain present in each.

### Data Availability

The data that support the findings of this study are available from the corresponding authors on request (datainquiryEXSS@imperial.ac.uk).

## Additional Information

**How to cite this article**: Shautsova, V. *et al*. Hexagonal Boron Nitride assisted transfer and encapsulation of large area CVD graphene. *Sci. Rep.* **6**, 30210; doi: 10.1038/srep30210 (2016).

## Supplementary Material

Supplementary Information

## Figures and Tables

**Table 1:** The position of the Dirac point and hole/electron mobility for graphene transistors measured after preparation and after 1 month of storage in ambient conditions.

sample	Dirac point as prepared (V)	mobility as prepared (cm^2^ V^−1^ s^−1^)		Dirac point after 1 month (V)	mobility after 1 month (cm^2^ V^−1^ s^−1^)	
		hole	electron		hole	electron
WT-Gr/SiO_2_	18	1069	331	26.5	872	301
WT-Gr/hBN	26	718	79	32.5	415	—
WT-Gr/SAM	12	380	170	30	288	95
AT-hBN/Gr	7	3105	836	9.6	1529	639
