# Supplementary material for: Hexagonal Boron Nitride assisted transfer and encapsulation of large area CVD graphene
Source: Sci Rep. 2016 Jul 22;6:30210. doi: 10.1038/srep30210 (PMC4957148; doi:10.1038/srep30210)
Supplement: Supplementary Information [file srep30210-s1.pdf]

Supplementary

“Hexagonal Boron Nitride assisted transfer and encapsulation of large area CVD graphene”

Viktoryia Shautsova*, Adam M. Gilbertson, Nicola C. Black, Stefan A. Maier, Lesley F. Cohen

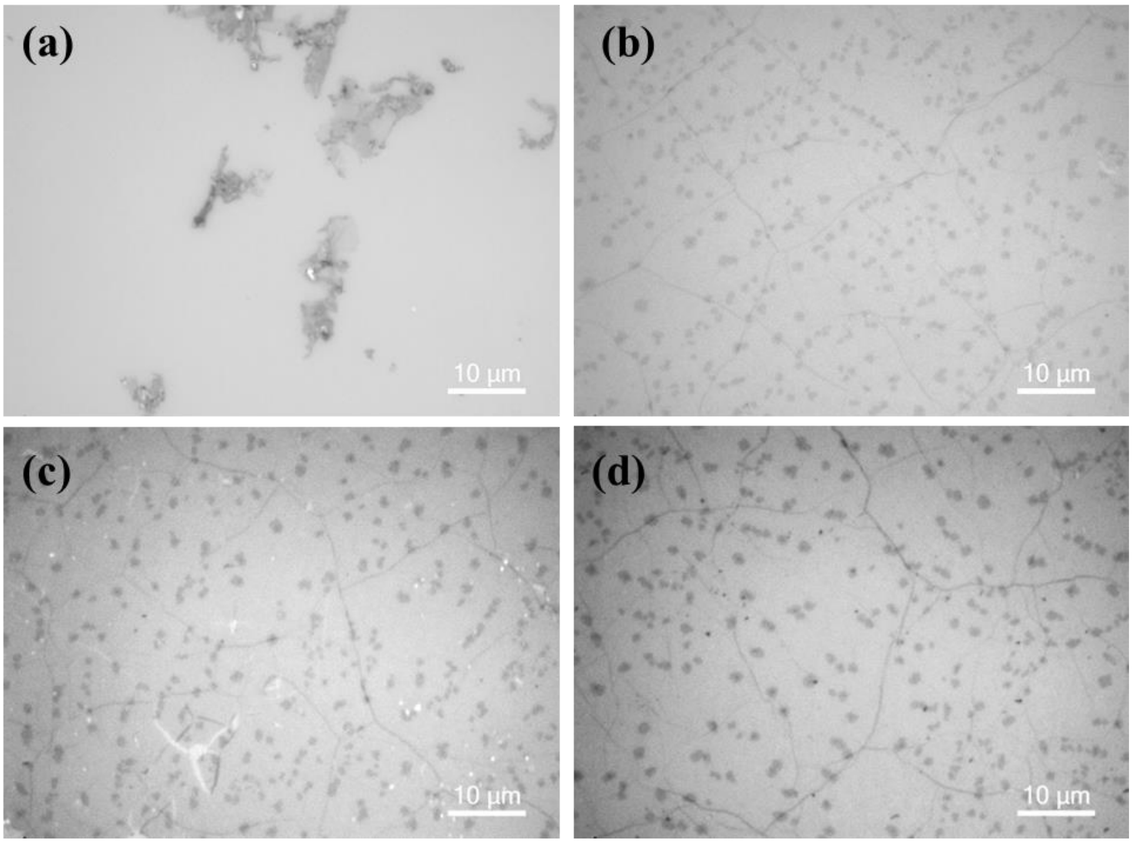

Fig. S1. Graphene transferred with dry transfer technique on untreated (a), O2-plama treated (b), SAM-modified (c) and hBN covered (d) SiO2 substrate. Only small-size graphene flakes are transferred in the case of the untreated SiO2 substrate.

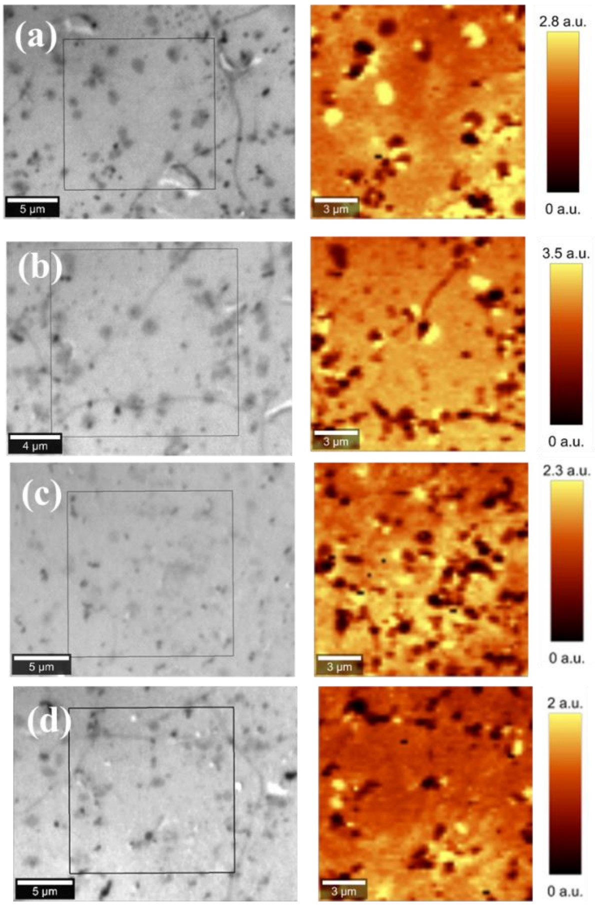

Fig. S2. The OM images (left) and corresponding micro-Raman images of the I(2D)/I(G) ratio (right) obtained for the samples transferred using wet transfer technique on untreated SiO2 (a), SAM- (b) and hBN- (c) covered SiO2 and using hBN-assisted transfer (d).

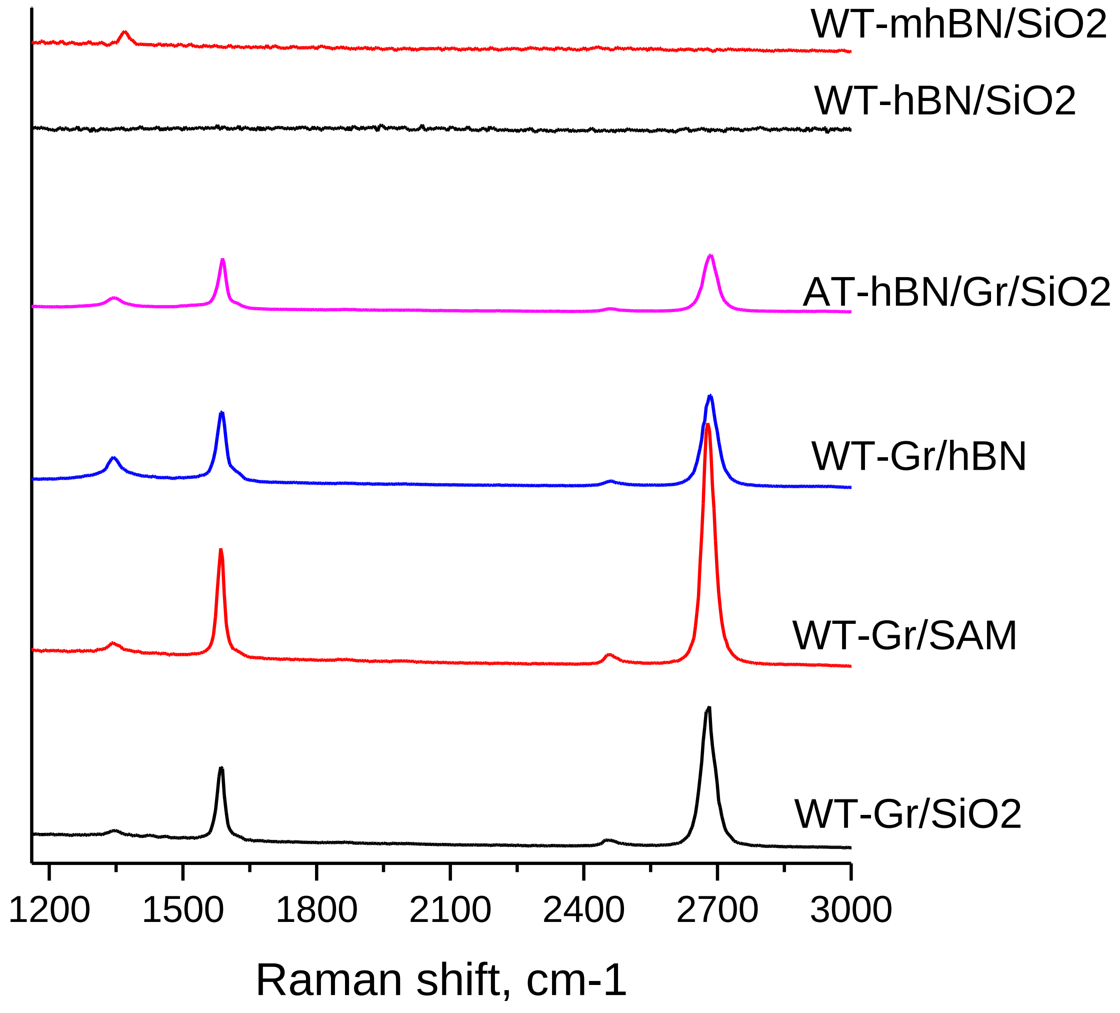

Fig. S3. Representative Raman spectra of the four samples discussed in the paper, namely AT-hBN/Gr/SiO_2, WT-Gr/hBN, WT-Gr/SAM and WT-Gr/SiO_2. The data is taken with 532 nm laser excitation at 1 mW power using a 0.5 second integration time. Under these conditions the spectrum of the single layer hBN on SiO_2/Si, WT-hBN/SiO_2, is also presented to confirm that the hBN peak is significantly weaker compared to graphene and cannot be resolved using these measurement protocols. On multilayer hBN with thickness around 50 nm, WT-mhBN/SiO_2, the known spectra of hBN is recovered.

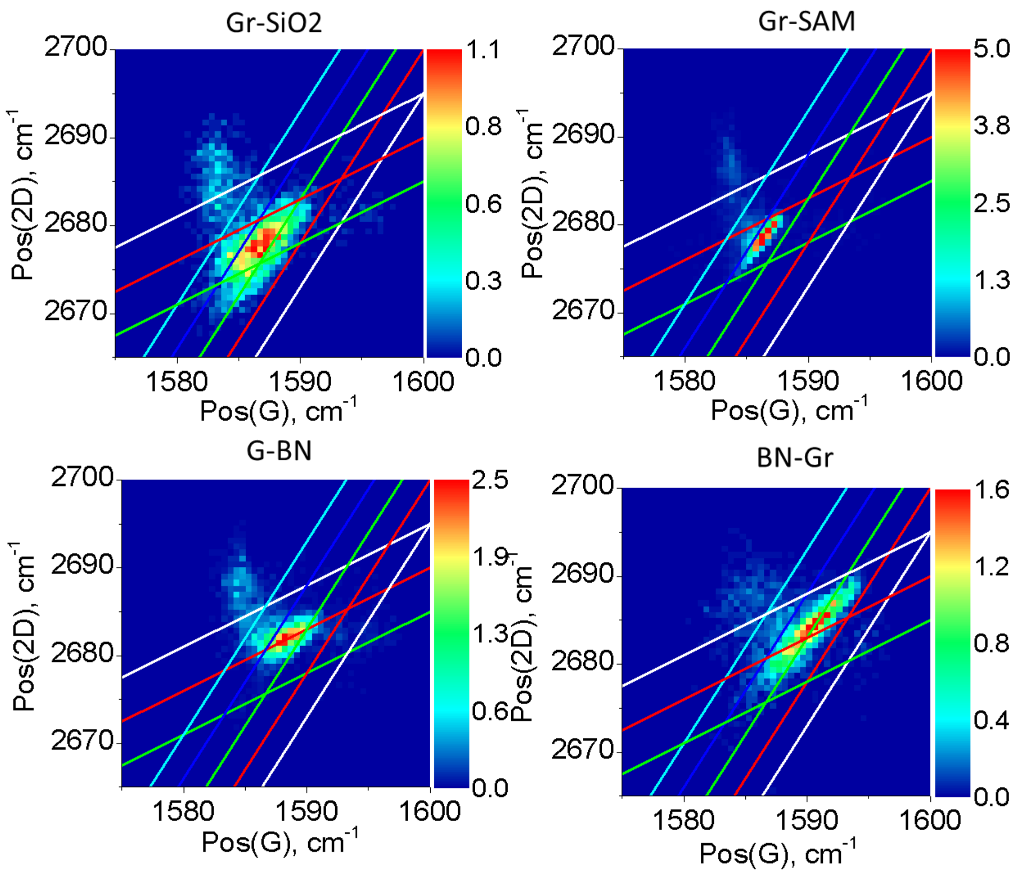

Fig. S4. 2D histograms with guide curves for doping and strain.

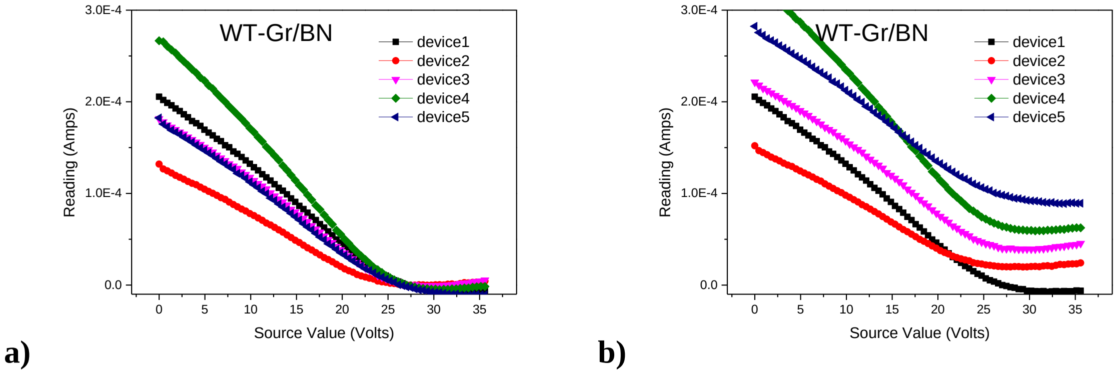

Fig. S5. The variation of transfer curves for the graphene FETs resulted from WT-Gr/BN film after 1 month storage in ambient conditions. The curves are superimposed to facilitate comparison of the hole mobility in each device **(a)** or offset, in order to facilitate the V_D comparison **(b).**

Device	V_D, V	hole mobility, $cm^2 V^{-1} s^{-1}$
device1	31.5	415.3
device2	32	278.4
device3	33.8	372.8
device4	28.8	556.6
device5	27.9	371.3
mean values	30.8±2.4	399±101

Table S1. The comparative results, the position of the Dirac point V_D and hole mobility, taken from the data shown in Fig. S5.
